# Alisol B 23-acetate induces autophagic-dependent apoptosis in human colon cancer cells via ROS generation and JNK activation

**DOI:** 10.18632/oncotarget.19605

**Published:** 2017-07-26

**Authors:** Yueliang Zhao, Edmund T.S. Li, Mingfu Wang

**Affiliations:** ^1^ School of Biological Sciences, The University of Hong Kong, Hong Kong, China

**Keywords:** alisol B 23-acetate, autophagy, apoptosis, ROS, JNK

## Abstract

Alisol B 23-acetate (AB23A), a natural triterpenoid from the rhizome of *Alisma orientale*, a Chinese medicinal herb, has multiple physiological activities including anticancer. However, its effect on human colon cancer and the underlying mechanism are not clear. Here, we reported for the first time that AB23A induced cell cycle G_1_ phase arrest and apoptotic cell death in colon cancer cells. Autophagy also occurred in AB23A-treated HCT116 cells as evidenced by the accumulation of microtubule-associated protein 1 light chain 3 form II (LC3-II) and degradation of SQSTM1/p62. An autophagy inhibitor, 3-methyladenine (3-MA) was found to attenuate AB23A-mediated autophagy, apoptosis, and cell death, indicating that AB23A-induced apoptotic response was dependent on the induction of autophagy. In addition, the treatment of HCT116 cells with AB23A resulted in the generation of reactive oxygen species (ROS) and phosphorylation of c-Jun N-terminal kinase (JNK). A ROS scavenger, N-acetylcysteine (NAC) and a JNK-specific inhibitor, SP600125 attenuated AB23A-induced autophagy and apoptotic cell death. Moreover, NAC was able to eliminate AB23A-induced JNK phosphorylation. This finding provides a novel mechanism of action of AB23A in colon cancer HCT116 cells that AB23A induces autophagic-dependent apoptotic cell death in colon cancer cells, at least in part, though the accumulation of intracellular ROS and subsequent activation of JNK.

## INTRODUCTION

**Figure 1:**
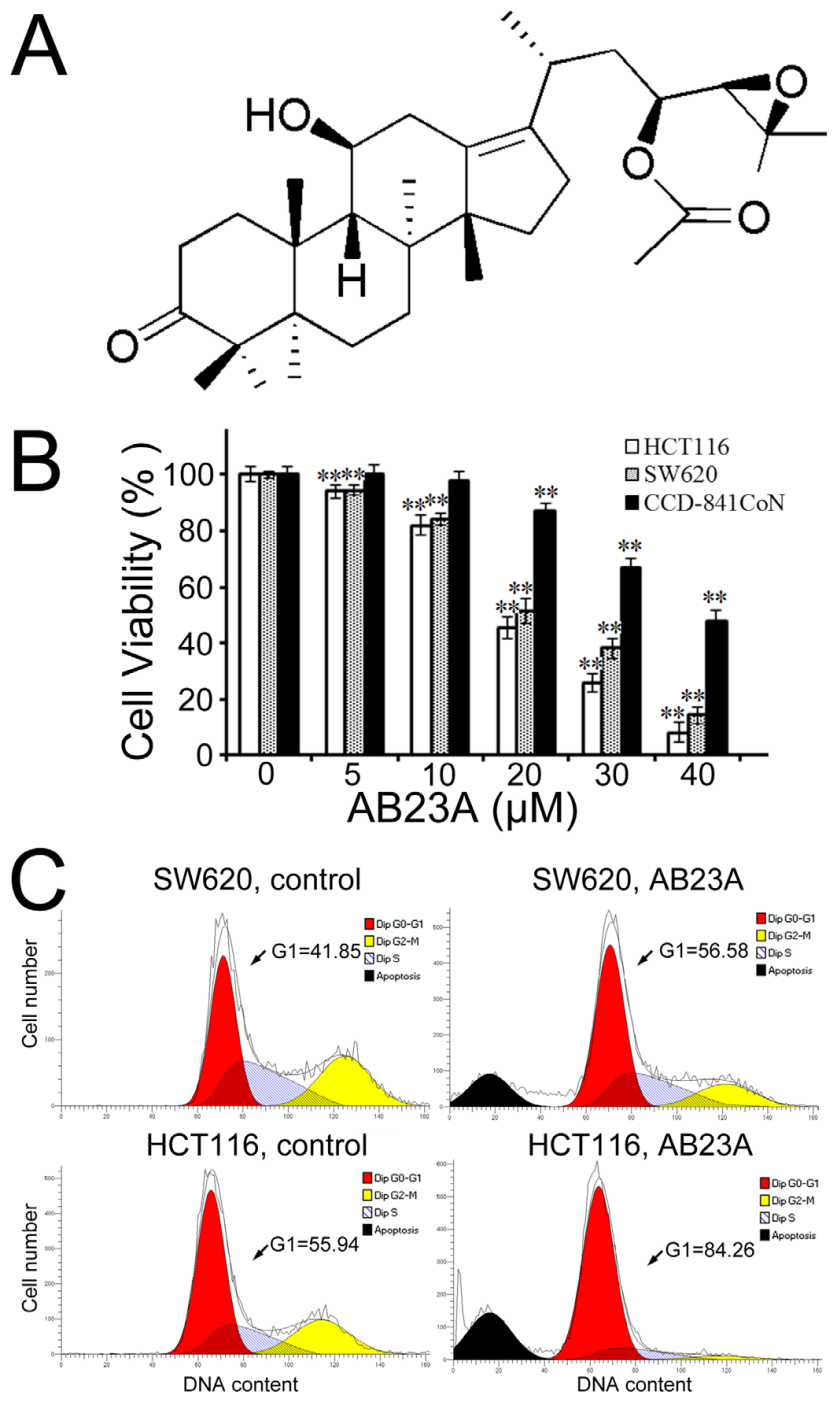
AB23A inhibits cell proliferation and induces cell cycle G_1_ phase arrest in human colon cancer cells **(A)** Chemical structure of AB23A. **(B)** Cell viability was determined after treatment with AB23A at various concentrations for 24 h. **(C)** SW620 and HCT116 cells, treated with vehicle (DMSO) or 20μM of AB23A for 24 h, were analyzed for DNA contents by flow cytometry. Values are mean ± standard deviation, n=3. * p<0.05, **p<0.01.

Natural products are important sources of novel anticancer agents and contribute a lot to cancer chemotherapy and chemoprevention [1]. Alisol B 23-acetate (AB23A) (Figure 1A) is a protostane-type triterpenoid isolated from *Alismatis Rhizoma* which is a medicinal plant formulated into different traditional Chinese herbal medicine formulas and widely used in the treatment of urological diseases and other aliments. In recent years, many studies have revealed that AB23A possesses several pharmacological activities, including anti-hepatitis virus [2], antibacterial [3], and hepatoprotective effect [4, 5]. Furthermore, AB23A has attracted great attention for its potential anticancer effects.

Apoptosis, as the type-I programmed cell death (PCD), is a mechanism exploited by many anticancer drugs to cause cancer cell death. AB23A has been demonstrated to induce apoptotic cell death in human hepatoma Hep3B cells, vascular smooth muscle A7r5 cells, human acute lymphoblastic leukemia CEM cells, and human hormone-resistant prostate cancer PC-3 cells [6–8]. Autophagy, a lysosomal catabolic pathway of self-degradation and recycling of cellular macromolecules and organelles, is often involved in the response to the treatment with anticancer agents [9, 10]. In some cellular settings, autophagy serves as a cell survival pathway suppressing apoptosis, and in others, it can lead to cancer cell death both in cells that are capable of apoptotic cell death and in cells that are deficient in apoptotic cell death [11]. Whether AB23A can induce apoptosis or autophagy in AB23A-treated colon cancer cells remains to be determined.

Reactive oxygen species (ROS), some active forms of oxygen, are generated as by-products of cellular metabolism, primarily in the mitochondria [12]. Some recent work has demonstrated that ROS function as second messengers participating into a wide variety of cell signaling pathways, including autophagy, apoptosis, gene expression, and the activation of cell signaling cascades, such as those involving mitogen-activated protein kinases (MAPK signal transduction cascades) [11, 13–15]. A moderate increase in ROS can promote cell proliferation and differentiation, whereas excessive cellular production of ROS can interfere with cellular signaling pathways by causing oxidative damage to cellular macromolecules such as lipids, proteins, and DNA [16–18]. Interestingly, some accumulating evidence suggests that cancer cells are frequently under increased burden of oxidative stress, and therefore more vulnerable to the damage promoted by further ROS insults induced by some exogenous agents [19]. Thus some chemical agents targeting of related signaling pathways, particularly the ROS/MAPK signaling, may be effective in the treatment of human cancers. However, the effect of AB23A-mediated ROS production and triggering of related signaling pathways in human colon cancer cells remain unclear.

Colon cancer is one of the most common malignancies worldwide. In the present study, we aimed to determine the anticancer activity of AB23A in human colon cancer SW620 and HCT116 cells. Cell viability assay, apoptosis assay, autophagy assay, ROS assay, western blot assay, and kinase inhibitors were employed to investigate the potential intracellular signal transduction pathways involved in AB23A-induced cell growth inhibition.

## RESULTS AND DISCUSSION

### AB23A inhibits cell proliferation in human colon cancer cells

Traditional Chinese herbal medicines have been considered as a rich source for the discovery of novel therapeutic agents with new structural features and mechanism of action due to thousands of years of history in clinical use. AB23A is a major ingredient isolated from the herb *Alismatis rhizoma* which has been used as a key ingredient in some traditional Chinese medicines for urological disease-related symptoms. In recent years, AB23A has been demonstrated to be an oral active component in the treatment of various kinds of diseases including nephritic syndrome, hemolysis, and allergy. It has also been reported that AB23A could induce cell apoptosis in leukemia and hepatoma cells [8]. Thus, AB23A has the potential to be a novel anticancer agent. In this study, we investigated the anticancer effect of AB23A in human colon cancer SW620 and HCT116 cells, which is the second leading cause of cancer death worldwide.

We first tested the effect of AB23A on cell proliferation in two human colon cancer cell lines. As shown in Figure 1B, after the exposure of SW620 and HCT116 cells to AB23A for 24 h, AB23A markedly inhibited the proliferation of SW620 and HCT116 in a dose-dependent manner with IC_50_ values of about 20μM in these two cancer cell lines. In contrast, AB23A exhibited much lower cytotoxicity towards a normal colonic epithelial cell line, CCD-841CoN at the same concentrations as were used on cancer cells (Figure 1B). In addition, AB23A showed strong cytotoxicity against human kidney cancer cell line HEK293T, human gastric cancer cell line AGS, and human liver cancer cell line PLC8024 (Supplementary Figure 1). These findings prompted us to further evaluate the activity as well as action mechanism of AB23A in the colon cancer cell lines.

### AB23A induces cell cycle G_1_ arrest in human colon cancer cells

The interference with cell cycle progression is one of the properties of many anticancer agents. AB23A has been shown to induce G_1_ phase cell cycle arrest in ovarian cancer cell lines before [20]. Thus the effect of AB23A on cell cycle profile of colon cancer cells was analyzed by flow cytometry in our study. As expected, the treatment with AB23A (20 μM) for 24 h was found to cause a substantial accumulation of cells at G_1_ phase in SW620 and HCT116 cells (Figure 1C), indicating that AB23A arrested SW620 and HCT116 cells at G_1_ phase of the cell cycle and subsequently blocked cell growth.

### AB23A induces apoptosis in SW620 and HCT116 cells

**Figure 2:**
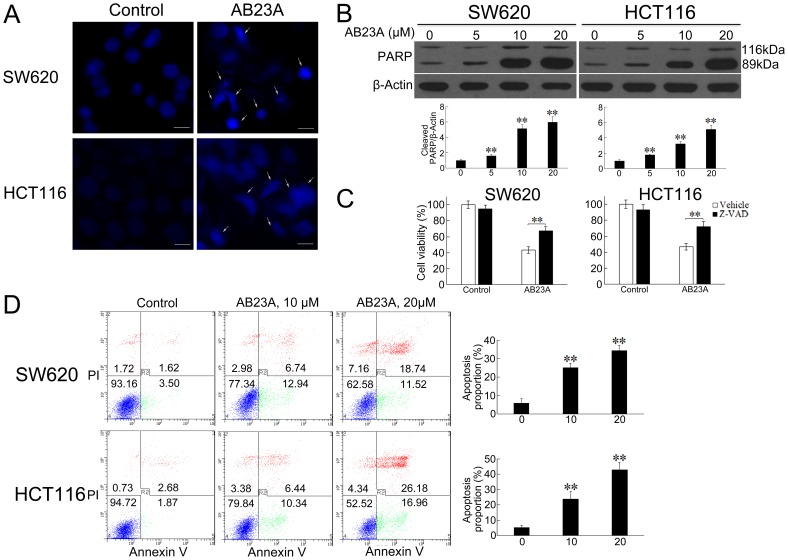
AB23A induces apoptosis in colon cancer cells **(A)** SW620 and HCT116 cells were treated with 20 μM of AB23A for 24 h, stained with Hoechst 33342 and photographed by fluorescence microscopy. Bar, 10 μm. Arrows show the apoptotic cells. **(B)** After AB23A treatment at indicated concentrations for 24 h, the effect of AB23A on the PARP cleavage was examined by Western blot. **(C)** Cell viability was assessed in SW620 and HCT116 cells treated for 24 h with AB23A (20 μM) in the presence or absence of Z-VAD (20 μM). **(D)** SW620 and HCT116 cells were treated with AB23A at indicated concentrations for 24 h, stained with Annexin V–FITC/PI, and analyzed by flow cytometry. Percentages of apoptotic cells are presented in the histogram. Values are mean ± standard deviation, n=3, **p<0.01.

In the past, accumulated data indicated that many anticancer drugs could induce apoptotic cell death in tumor cells. As AB23A has a steroid-like structure, it is expected that AB23A possesses some steroid-related pharmacological activities, such as glucocorticoid-mediated apoptosis in murine T cell number and function [21]. As expected, the PI staining of cells treated with AB23A showed remarkable increased proportion of sub-G_0_/G_1_ cells (Figure 1C), indicating the induction of apoptosis in response to AB23A treatment. The induction of colon cancer cell apoptosis was also observed by employing Hoechst 33342 staining. The results showed that AB23A elicited appreciable apoptotic response in SW620 and HCT116 cells, as evidenced by the appearance of cells with nuclear condensation (Figure 2A). This was further confirmed by analyzing the expression of cleaved PARP. As shown in Figure 2B, cleaved PARP was observed notably in cells treated with AB23A for 24 h. Moreover, a pan-caspase inhibitor, Z-VAD-FMK (Z-VAD) decreased cell death induced by AB23A (Figure 2C) significantly. Hence, the cytotoxic effect of AB23A in human colon cancer SW620 and HCT116 cells is likely due to the induction of apoptosis. To quantify the apoptosis, cells treated with AB23A were stained with Annexin V–FITC/PI and analyzed by flow cytometry. the exposure of cells to AB23A for 24 h resulted in a significantly increased percentage of apoptotic cells (Figure 2D). Collectively, these results suggested that AB23A exhibited potential anticancer activity, at least in part, by inducing apoptosis in colon cancer cells.

### Excessive autophagy induced by AB23A leads to apoptotic cell death in HCT116 cells

**Figure 3:**
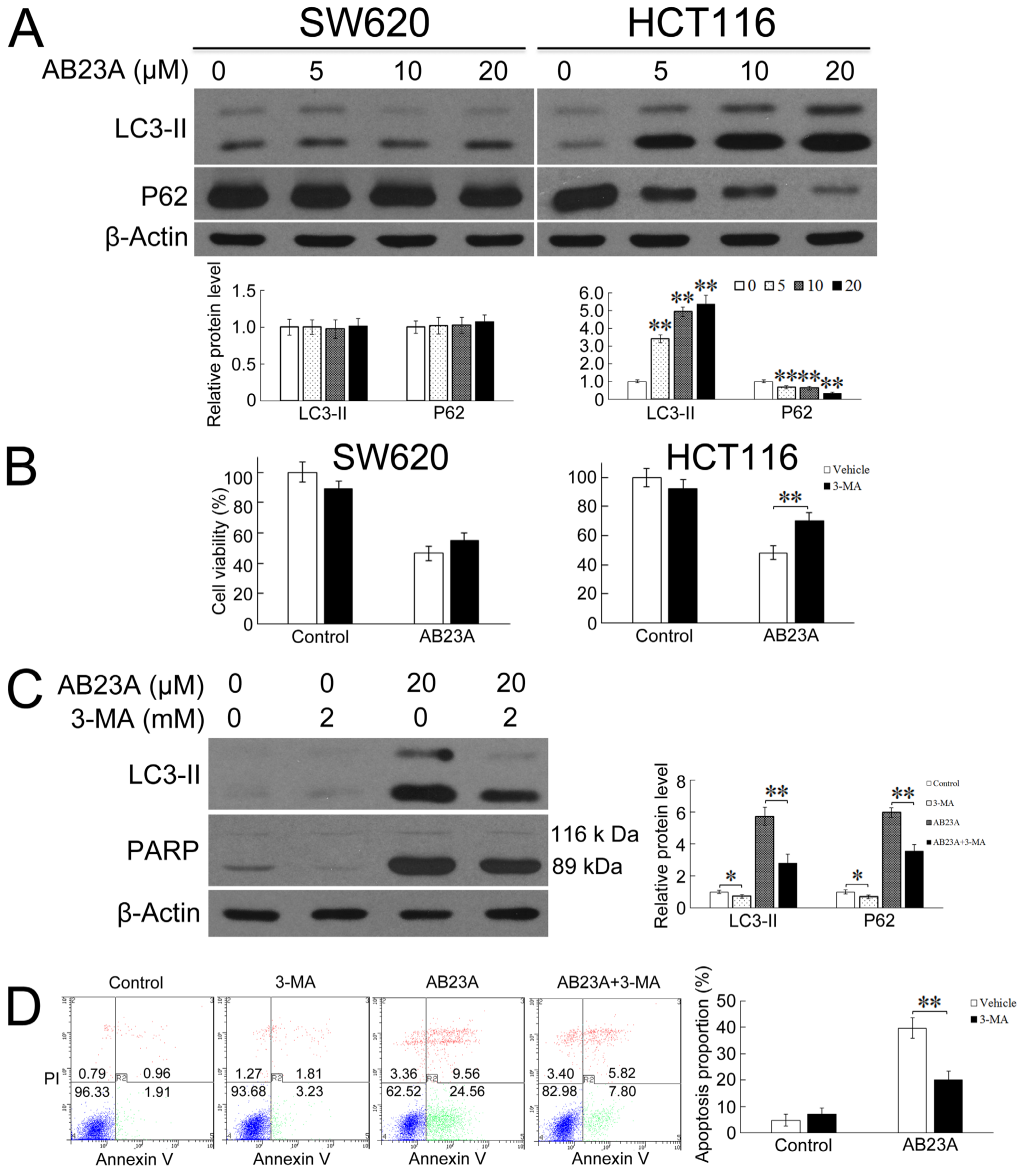
Inhibition of autophagy protects HCT116 cells from AB23A-induced cell death and apoptosis **(A)** SW620 and HCT116 cells were exposed to AB23A for 24 h at various concentrations. Then expression levels of LC3-I, LC3-II, and P62 were assessed by immunoblot analysis. **(B)** HCT116 cells were treated with AB23A (20 μM) in the absence or presence of 2 mM of 3-MA for 24h. Then cell viability was assessed by the cck-8 assay, **(C)** expression levels of LC3-I, LC3-II, and cleaved PARP were assessed by immunoblot analysis, and **(D)** the percentage of apoptosis was determined by flow cytometry. Values are mean ± standard deviation, n=3, *p<0.05, **p<0.01.

We next asked whether AB23A induced autophagy in SW620 and HCT116 cells. Microtubule-associated protein 1 light chain 3 (LC3) is a mammalian protein that mainly exists in two forms, LC3-I and LC3-II. LC3-I in the cytosol is converted to the membrane-bound LC3-II during autophagosome formation. The levels of LC3-II have been shown to correlate with the number of autophagosomes per cell [22]. The increased production of LC3-II has been used as indicators for autophagy induction [23]. Thus, we measured the expression levels of LC3-II by immunoblot assays. As shown in Figure 3A, the treatment of cells with AB23A resulted in significant increases of LC3-II level in HCT116 cells in a dose-dependent manner. However, the treatment with AB23A did not change the expression of LC3-II in SW620 cells. To further clarify the ability of AB23A to induce autophagy, we measured the expression of SQSTM1/p62, which is a selective substrate of autophagy [24]. In line with the enhanced LC3 turnover, p62 expression level was significantly decreased after AB23A treatment in HCT116 cells. These results strongly supported the induction of autophagy by AB23A in HCT116 colon cancer cells.

The induction of autophagy and apoptosis in HCT116 cells in response to AB23A treatment raised the question whether these two events were linked or independent events. When HCT116 cells were co-treated with AB23A and an autophagy inhibitor, 3-MA, cell viability was markedly improved in comparison with cells treated with AB23A alone (Figure 3B), suggesting that AB23A-induced HCT116 cell death was dependent on autophagy. Further, the treatment of cells with 3-MA resulted in not only a marked reduction of the level of LC3-II, but also a reduction of cleaved PARP and percentage of apoptotic cells in AB23A-treated cells (Figure 3C and 3D), suggesting that the ability of AB23A to elicit an apoptotic response was dependent on induction of autophagy. Collectively, AB23A induced autophagic-dependent apoptotic cell death.

### AB23A induces autophagy and apoptosis via ROS generation in HCT116 cells

**Figure 4:**
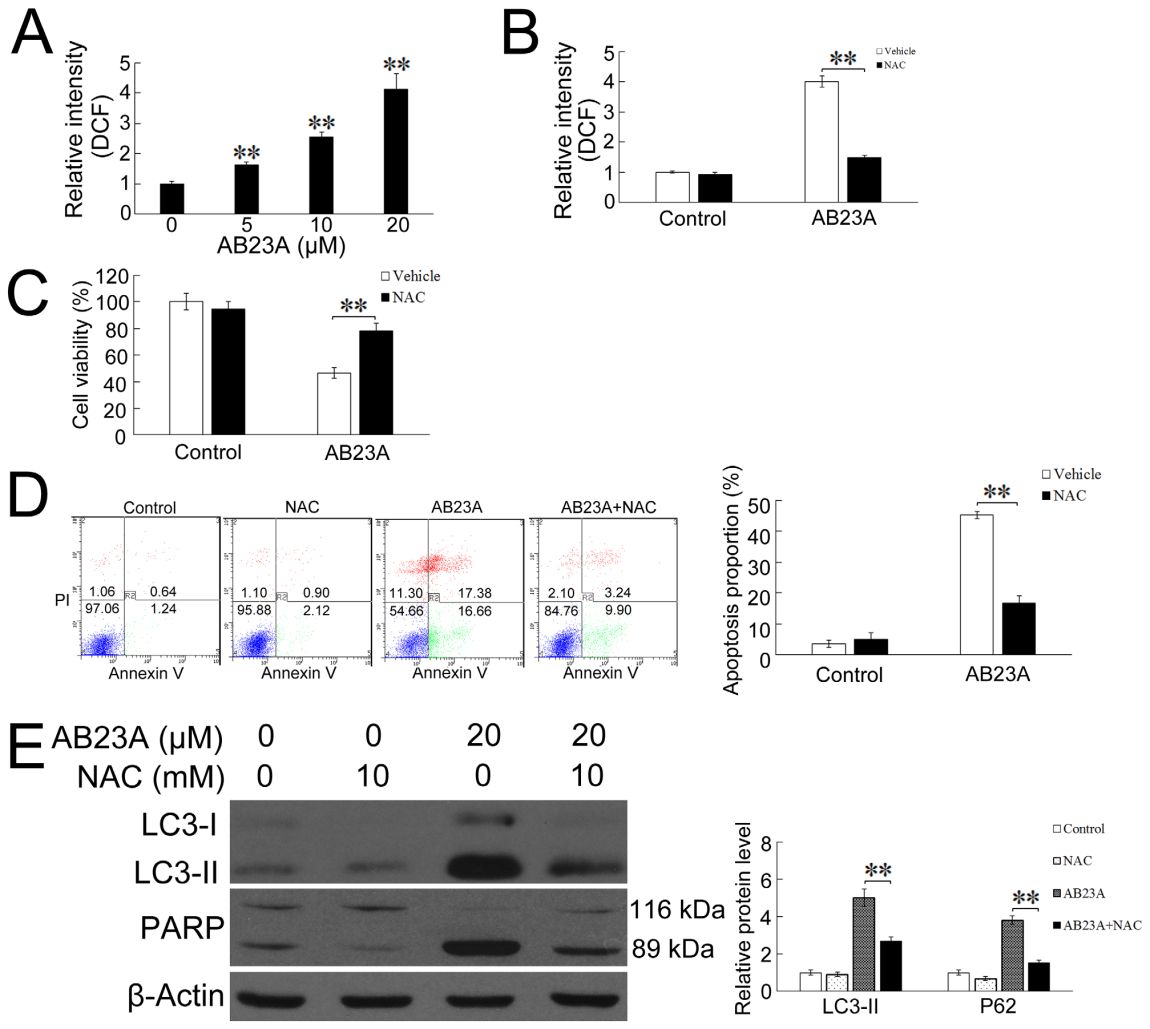
ROS are involved in AB23A-induced autophagy and apoptosis **(A)** HCT116 cells were treated with AB23A at various concentrations for 6 h, ROS levels were measured as DCF fluorescence intensity by flow cytometry. **(B)** HCT116 cells were pretreated with the ROS scavengers NAC (10 mM) for 1 h and then AB23A (20 μM) was included in the incubation for 6 h. ROS levels were measured by flow cytometry. **(C)** HCT116 cells were pre-incubated with NAC (10 mM) for 1 h, and then treated with AB23A (20 μM) for 24 h. Cell viability was measured by cck-8 kit. **(D)** Cells were treated with AB23A and NAC as in (C), before the percentage of apoptosis was determined by flow cytometry. **(E)** Cells were treated with AB23A and NAC as in (C), then expression levels of LC3-I, LC3-II, and cleaved PARP were measured by Western blot. Values are mean ± standard deviation, n=3, **p<0.01.

The deregulation of cellular redox balance plays a pivotal role in the pro-apoptotic activities of various anticancer agents [25, 26]. The overproduction of ROS and free radicals can cause serious damage to lipids, proteins, and DNA, and regulate the process involved in initiating autophagy and apoptosis [27, 28]. Recently, it has been reported that ROS might be involved in steroid-induced apoptosis of lymphocytes [29, 30]. We therefore tried to determine whether AB23A-induced cytotoxicity was associated with the production of intracellular ROS. As shown in Figure 4A, AB23A caused a dose-dependent increase of the level of intracellular ROS in HCT116 cells. The pre-treatment with a ROS scavenger, NAC for 1h significantly attenuated AB23A-induced ROS production and reversed AB23A-mediated cell growth inhibition (Figure 4B and 4C), suggesting AB23A elicited cytotoxicity through ROS generation. The mitochondria and NADPH oxidase system were considered as the major producers of ROS within the cells [12, 31, 32]. We next studied the effects of a mitochondria-targeted antioxidant, MitoTEMPOL (MitoT) and a NADPH oxidase inhibitor, apocynin on the AB23A-induced ROS generation and cytotoxicity. The results showed that MitoTEMPOL, but not apocynin, significantly attenuated AB23A-induced ROS production as well as inhibitory effect on HCT116 cell growth (Supplementary Figure 2), suggesting AB23A induced ROS production via mitochondria pathway.

To clarify the role of ROS in mediating AB23A-induced autophagy and apoptosis, we further analyzed the percentage of apoptotic cells and the expression level of LC3-II and cleaved PARP in the presence of NAC. As shown in Figure 4D, NAC significantly blocked AB23A induced apoptosis. In addition, Western blot analysis showed that NAC suppressed the increased expression of LC3-II and cleaved PARP induced by AB23A (Figure 4E). Collectively, these data provide convincing evidence that AB23A-induced autophagic-dependent apoptotic cell death through ROS generation.

### JNK activation is involved in AB23A-induced autophagy and apoptosis

**Figure 5:**
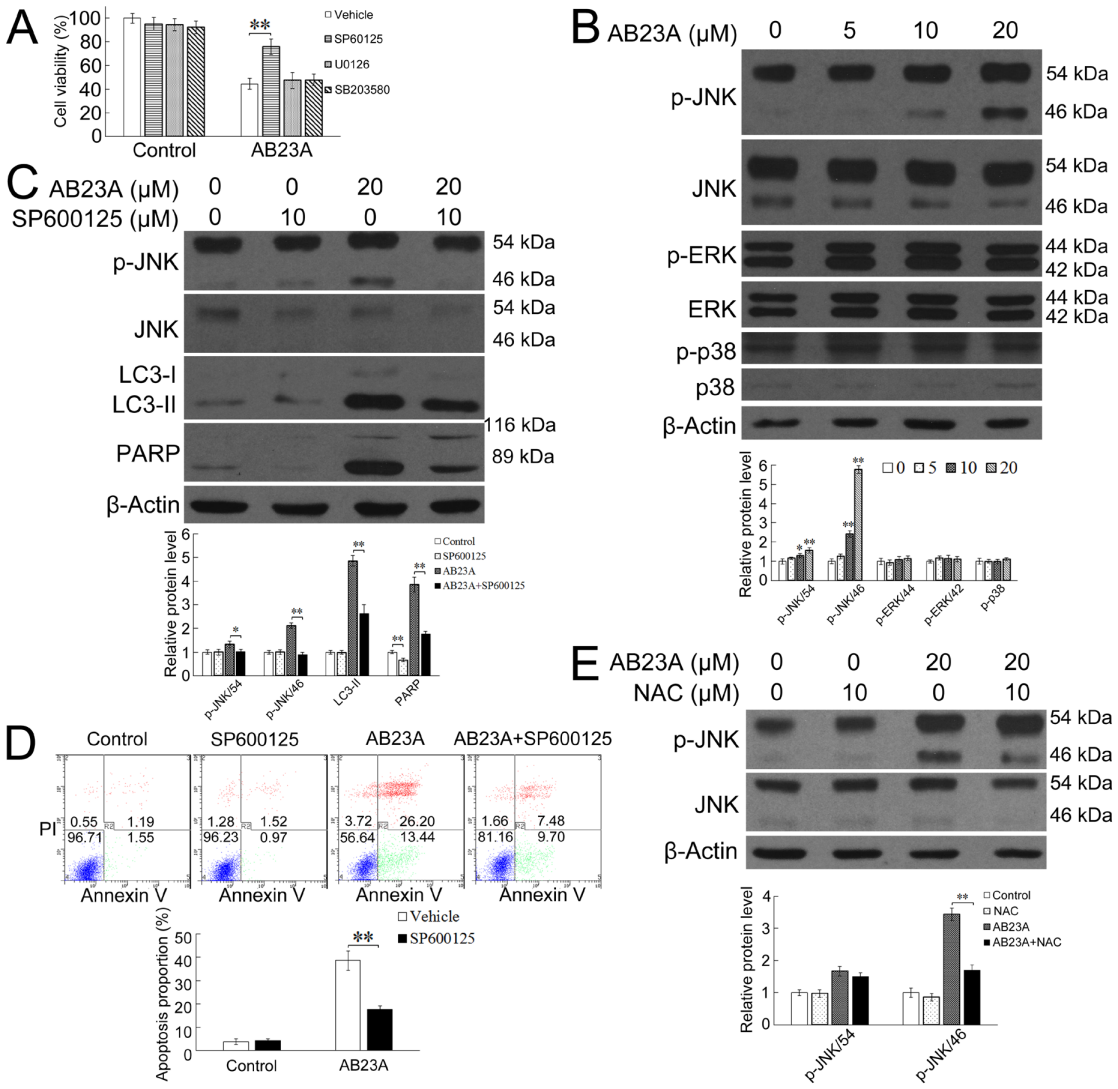
ROS generation is upstream of JNK activation and JNK activation is required for the autophagic-dependent apoptotic cell death induced by AB23A **(A)** Cell viability of HCT116 cells treated with AB23A (20 μM) for 24 h in the presence or absence of the MEK1/2 inhibitor U0126 (10 μM), the JNK inhibitor SP600125 (10 μM), or the p38 inhibitor SB203580 (10 μM). **(B)** Western blot analysis of p-JNK, JNK, p-ERK, ERK, p-p38, p38, and β-actin in HCT116 cells treated with AB23A at indicated concentrations for 24 h. **(C)** HCT116 cells were treated with AB23A (20 μM) for 24 h in the presence or absence of SP600125 (10 mM). Then the expression levels of phospho-JNK, JNK, LC3-II, and PARP were analyzed by Western blot assay and **(D)** the percentage of apoptotic cells was measured by flow cytometry. **(E)** Western blot analysis of p-JNK and JNK levels in HCT116 cells treated with AB23A (20 μM) for 24 h in the presence or absence of NAC (10 mM). Values are mean ± standard deviation, n=3, *p<0.05, **p<0.01.

The MAPK pathways which consist of three main members, the extracellular signal-regulating kinase (ERK), c-Jun N-terminal protein kinase (JNK), and p38 has been documented to play important roles in autophagy and apoptosis regulation [33, 34]. The oleanane triterpenoid, 2-cyano-3,12-dioxoolean-1,9-dien-28-oic acid (CDDO), its C-28 methyl ester (CDDOMe) and C-28 imidazolide ester (CDDO-Im) derivatives-induced apoptosis in ML-1 and U-937 leukemia cells has been demonstrated to be associated with JNK activation [35]. Having established that the apoptotic cell death induced by AB23A required ROS generation, we then asked whether AB23A-induced apoptosis required MAPK activation. HCT116 cells were thus exposed to AB23A for 24 h together with or without various MAP kinase inhibitors that blocked a specific signaling pathway leading to cell death. The JNK inhibitor SP600125, but not the p38 inhibitor SB203580, nor the ERK inhibitor U0126, could partially rescue the loss of cell viability caused by AB23A (Figure 5A), suggesting that AB23A likely induced cell death via the JNK signaling pathway. JNK, ERK, and p38 MAPK activation in response to AB23A were then examined in HCT116 cells. As shown in Figure 5B, AB23A increased the level of phosphorylated JNK, while having a minimum effect on the expression of phosphorylated ERK and p38, substantiating the involvement of JNK-associated signaling pathway in AB23A-induced colon cancer cell death. We then asked whether AB23A-induced autophagy and apoptosis requires JNK activation. As shown in Figure 5C and 5D, the JNK inhibitor SP600125 significantly attenuated LC3-II and cleaved PARP level and the percentage of apoptotic cells in response to AB23A treatment, indicating that the JNK pathway was involved in AB23A-induced autophagy and apoptosis.

ROS has been reported to be a potent regulator of MAP kinase family members and subsequent cell death [35, 36]. The above observation confirmed that AB23A-induced autophagy and apoptotic cell death is dependent on intracellular ROS generation and activation of JNK. To further elucidate the relationship between ROS and JNK in AB23A-induced cell death, the effect of NAC on JNK activation was investigated. As shown in Figure 5E, NAC markedly suppressed AB23A-induced JNK phosphorylation, suggesting ROS is upstream of JNK activation in AB23A-induced cell death. Taken together, these results suggested that AB23A induced autophagic-dependent apoptotic cell death in human colon cancer cells via ROS generation and subsequent JNK activation

**Figure 6:**
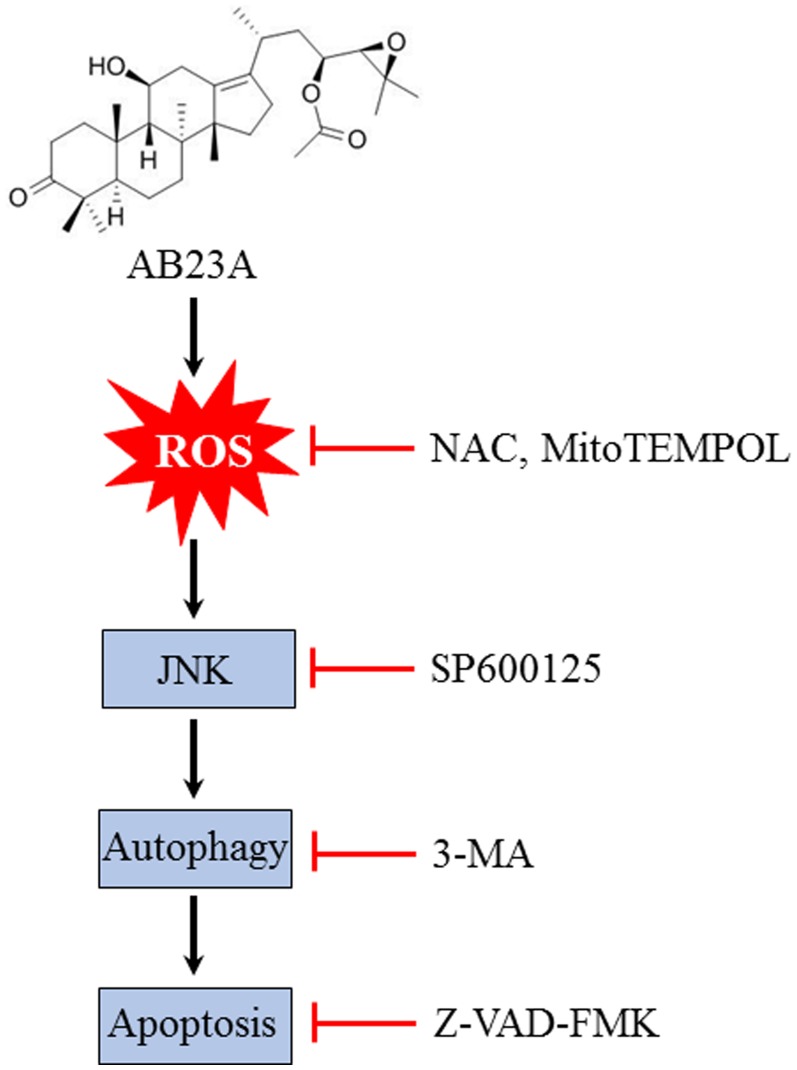
Schematic illustration of the mechanisms of AB23A-induced autophagy and apoptosis in HCT116 cells

Collectively, we have revealed that AB23A, a protostane-type triterpenoid from the Chinese herb *Alisma orientale* elicited potent anticancer effect in human colon cancer cells by inducing cell cycle arrest and autophagic-dependent apoptotic cell death. Induction of ROS generation and subsequent activation of JNK may account for the pharmacological effects of AB23A (Figure 6). AB23A may be a promising molecule in colon cancer treatment which merits further investigation.

## MATERIALS AND METHODS

### Chemical reagents and antibodies

2′,7′-Dichlorodihydrofluorescin diacetate (DCFH-DA) (sc-359840), N-acetyl-L-cysteine (NAC) (sc-202232), Z-VAD-FMK (sc-3067) was obtained from Santa Cruz. 3-methyladenine (3-MA) (M9281), U0126 monoethanolate (U120), SP600125 (s5567), and SB203580 (S8307) were purchased from Sigma. Primary antibodies against p-ERK1/2 Thr^202^/Tyr^204^ (4370), ERK1/2 (9102), p-JNK (9251), JNK (9252), p-p38 (4511), p38 (8690), PARP (9541), β-Actin (4967), and anti-mouse (7076) and anti-rabbit (7074) HRP conjugated secondary antibodies were obtained from Cell Signaling Technology. AB23A was obtained from National Institutes for Food and Drug Control (111846) with a purity of ≥ 98%. MitoTEMPOL (ab144644) was purchased from abcam. Apocynin (C10076750) was obtained from Macklin.

### Cell culture and cell viability assay

Human colon cancer cell lines SW620 and HCT116, human normal colon epithelial cell line CCD-841CoN, human kidney cancer cell line HEK293T, human gastric cancer cell line AGS, and human liver cancer cell line PLC8024 were purchased from the American Type Culture Collection (ATCC). The cells were maintained in DMEM (GIBCO, 12100-061) containing 10% fetal bovine serum (GIBCO, 10270) at 37°C under 5% carbon dioxide. CCD-841CoN cells were cultured in minimum essential medium (MEM) (GIBCO, 11095-080) with 10% fetal bovine serum (FBS) under the same conditions for the colon cancer cells. Cell viability was measured using a cell counting kit (cck-8) (Dojindo, CK-04) according to the manufacturer’s instructions. Briefly, cells (1×10^4^ cells/well) were seeded in 96-well microtiter plates overnight, treated with the respective agents for 24 h, and then incubated with cck-8 solution (10%) dissolved in the culture medium for 1 h at 37°C. Absorbance was measured at 450 nm on a Benchmark Plus microplate reader.

### Cell cycle analysis

Cells were treated with AB23A (25 μM) or vehicle for 24 h, harvested and fixed with cold ethanol (75%) at -20°C overnight. Cells were then washed twice with ice-cold PBS and stained with 50 μg/mL of propidium iodide (PI) (Sigma-Aldrich, P4170) in PBS containing 0.1% TritonX-100 (Sigma-Aldrich, T8787) and 100 μg/mL of RNAse-A (Invitrogen, 12091021) for at least 30 min in the dark. The cells were then analyzed using a flow cytometer (BD FACSAria III).

### Apoptosis assay

Apoptotic chromatin condensation was examined by fluorescent microscopy using Hoechst 33342 (0.5 μg/ml, Sigma-Aldrich, B2261) according to some previous studies [9, 10]. In brief, cells were exposed to the respective agents for 24 h and then incubated with Hoechst 33342 at 37°C for 30 min. After staining, the cells were washed three times with PBS, fixed in 4% paraformaldehyde, and then observed under a fluorescence microscope.

Apoptotic cells were quantified by Annexin V-FITC Apoptosis Detection Kit I (556547, BD Pharmingen) using flow cytometry according to the protocol provided. Briefly, cells were treated with the respective agents for 24 h and then washed with PBS and re-suspended in the binding buffer. After being incubated with annexin V–FITC (5 μL) and PI (10 μL, 50 μg/mL in PBS) mixture in the binding buffer (100μL) for 15 min at room temperature in the dark, 400 μL of binding buffer was added to each tube. The cells were then immediately analyzed on a flow cytometer (BD FACSAria III).

### Intracellular ROS generation assay

Intracellular ROS generation was measured by a ROS assay with DCFH-DA using flow cytometry according to a previous study [37]. Briefly, cells were plated on a six-well plate at a density of 5×10^5^ cells/well. After treatment with the respective agents for 6 h, the cells were washed with PBS and incubated with 10 μM DCFH-DA for 30 min at 37°C. The cells were then collected, washed with PBS, and analyzed immediately using a flow cytometer (BD FACSAria III). The mean fluorescence intensity of 10,000 cells was analyzed by BD FACSDIVA software.

### Western blotting assays

Cells (5×10^5^ cells/well) were seeded on a six-well plate overnight, and exposed to the respective agent for 24 h. Following the exposure, the cells were rinsed with PBS and lysed in RIPA buffer (89900, Thermo Scientific) containing protease inhibitor cocktail (Sigma, P8340), phosphatase inhibitor cocktail 2 (Sigma-Aldrich, P5726) and phosphatase inhibitor cocktail 3 (Sigma-Aldrich, P0044). Protein concentration was quantified using a Bio-Rad protein assay kit (Bio-Rad, 500-0006). Lysate proteins (10 μg) were separated on a 10% or 12% sodium dodecyl sulfate (SDS)–polyacrylamide gel and then electrophoretically transferred onto polyvinylidene difluoride (PVDF) membranes (Bio-Rad, 162-0177). The membranes were blocked with 5% nonfat milk or BSA in TBST for 1 h at room temperature and then incubated with the respective primary antibodies at 4°C overnight. After being washed five times with TBST, the membranes were incubated with the appropriate horseradish peroxidase-conjugated secondary antibodies for 1 h at room temperature. Protein bands were visualized by chemiluminescence detection kit (Thermo Fisher Scientific, 1856135). Densitometric quantification was performed using ImageJ software (National Institute of Health, Bethesda, MD, USA). β-Actin served as a loading control.

### Statistical analysis

All experimental data were the average of at least 3 independent tests. Differences between experimental groups were calculated using Student’s t-test. A p-value less than 0.05 was considered as statistically significant.

## SUPPLEMENTARY MATERIALS FIGURES


