# Supplementary material for: Alisol B 23-acetate induces autophagic-dependent apoptosis in human colon cancer cells via ROS generation and JNK activation
Source: Oncotarget. 2017 Jul 26;8(41):70239–49. doi: 10.18632/oncotarget.19605 (PMC5642550; doi:10.18632/oncotarget.19605)
Supplement: Supplementary file 1 [file oncotarget-08-70239-s001.pdf]

Alisol B 23-acetate induces autophagic-dependent apoptosis in human colon cancer cells via ROS generation and JNK activation

SUPPLEMENTARY MATERIALS

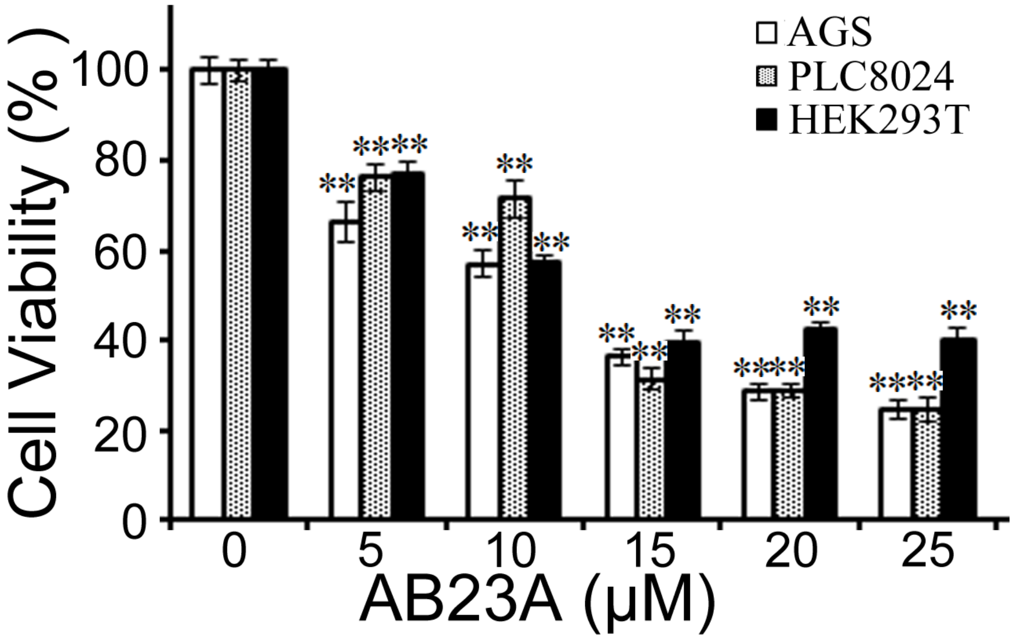

Supplementary Figure 1: AB23A inhibits cell proliferation in human kidney cancer cell line HEK293T, human gastric cancer cell line AGS, and human liver cancer cell line PLC8024. Cell viability was determined after treatment with AB23A at various concentrations for 24 h. Values are mean ± standard deviation, n=3. * $p<0.05$, ** $p<0.01$.

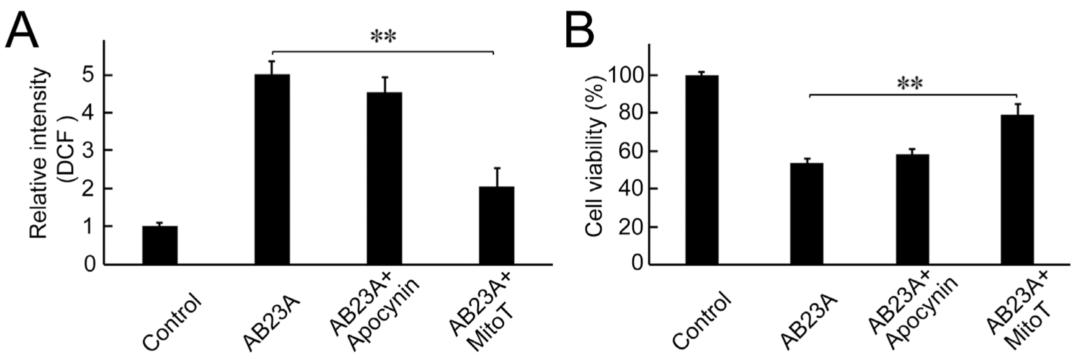

Supplementary Figure 2: AB23A induced ROS production via mitochondria pathway. (A) HCT116 cells were treated with AB23A (20 µM) for 24 h in the presence or absence of MitoTEMPOL (5 µM) or apocynin (50 µM). Then (A) ROS levels were measured by flow cytometry and **(B)** cell viability was measured by the cck-8 assay. Values are mean ± standard deviation, n=3. **p<0.01.
